# Moderate hypofractionation remains the standard of care for whole-breast radiotherapy in breast cancer: Considerations regarding FAST and FAST-Forward

**DOI:** 10.1007/s00066-020-01744-3

**Published:** 2021-01-28

**Authors:** David Krug, René Baumann, Stephanie E. Combs, Marciana Nona Duma, Jürgen Dunst, Petra Feyer, Rainer Fietkau, Wulf Haase, Wolfgang Harms, Thomas Hehr, Marc D. Piroth, Felix Sedlmayer, Rainer Souchon, Vratislav Strnad, Wilfried Budach, Breast Cancer Expert Panel of the German Society of Radiation Oncology (DEGRO)

**Affiliations:** 1 Department of Radiation Oncology, University Hospital Schleswig-Holstein, Arnold-Heller-Str. 3, 24105 Kiel, Germany; 2 Department of Radiation Oncology, St. Marien-Krankenhaus, Siegen, Germany; 3 Department of Radiation Oncology, Technische Universität München (TUM), Munich, Germany; 4 Department of Radiation Medicine (IRM), Helmholtz Zentrum München (HMGU), Neuherberg, Germany; 5 Partner Site Munich, Deutsches Konsortium für Translationale Krebsforschung (dktk), Munich, Germany; 6 Department of Radiation Oncology, University Hospital Jena, Jena, Germany; 7 Vivantes Hospital Neukölln, Berlin, Germany; 8 University Hospital Erlangen, Erlangen, Germany; 9 Formerly St.-Vincentius-Hospital, Karlsruhe, Germany; 10 St. Claraspital, Basel, Switzerland; 11 Department of Radiation Oncology, Marienhospital Stuttgart, Stuttgart, Germany; 12 HELIOS University Hospital Wuppertal, Witten/Herdecke University, Wuppertal, Germany; 13 Paracelsus Medical University Hospital, Salzburg, Austria; 14 Formerly University Hospital Tuebingen, Tuebingen, Germany; 15 Department of Radiation Oncology, University Hospital Düsseldorf, Düsseldorf, Germany

**Keywords:** Breast cancer, Radiotherapy, Hypofractionation, Local recurrence, Late toxicity

## Abstract

Moderate hypofractionation is the standard of care for adjuvant whole-breast radiotherapy after breast-conserving surgery for breast cancer. Recently, 10-year results from the FAST and 5‑year results from the FAST-Forward trial evaluating adjuvant whole-breast radiotherapy in 5 fractions over 5 weeks or 1 week have been published. This article summarizes recent data for moderate hypofractionation and results from the FAST and FAST-Forward trial on ultra-hypofractionation. While the FAST trial was not powered for comparison of local recurrence rates, FAST-Forward demonstrated non-inferiority for two ultra-hypofractionated regimens in terms of local control. In both trials, the higher-dose experimental arms resulted in elevated rates of late toxicity. For the lower dose experimental arms of 28.5 Gy over 5 weeks and 26 Gy over 1 week, moderate or marked late effects were similar in the majority of documented items compared to the respective standard arms, but significantly worse in some subdomains. The difference between the standard arm and the 26 Gy of the FAST-Forward trial concerning moderate or marked late effects increased with longer follow-up in disadvantage of the experimental arm for most items. For now, moderate hypofractionation with 40–42.5 Gy over 15–16 fractions remains the standard of care for the majority of patients with breast cancer who undergo whole-breast radiotherapy without regional nodal irradiation after breast-conserving surgery.

## Introduction

Moderate hypofractionation with 15–16 fractions of 2.6–2.7 Gy has been accepted as the standard of care for whole-breast external beam radiotherapy (EBRT) for invasive breast cancer in many countries [1–3]. This was based on the results from several well-powered randomized controlled trials showing comparable outcomes with regard to the risk of recurrence and chronic toxicity and with potential advantages in terms of reduced acute toxicity and improved cost-effectiveness [4–12]. First results on hypofractionated post-mastectomy radiotherapy have been published [13–15] with multiple trials on this topic and on hypofractionated regional nodal irradiation still ongoing. While there may be residual areas of debate, such as very young patients, rare histologic subtypes or patients with connective tissue diseases [16], there is now a broad consensus that moderate hypofractionation should be used preferentially after breast-conserving surgery when regional nodal irradiation is not indicated.

Boost irradiation was given sequentially with 5–8 fractions of 2 Gy in the START trials [6, 7] which led to prolongation of overall treatment time of 1–1.5 weeks. Since then, numerous trials have studied moderately hypofractionated radiotherapy with a simultaneous integrated boost (for review see [17]). However, oncological outcome results from two large randomized controlled phase III trials are still pending (RTOG 1005 [NCT01349322] and HYPOSIB [NCT02474641]). There are a number of reports of intraoperative boost irradiation for patients with breast cancer (for review see [18]). However, few trials studied the combination of hypofractionated whole-breast radiotherapy and intraoperative boost irradiation. First results from the prospective single-arm HIOB trial studying intraoperative boost irradiation with electrons followed by hypofractionated whole-breast radiotherapy have been published recently [19]. With a median follow-up of 45 months and 583 patients, toxicity rates and cosmetic outcome were favorable [19]. Regarding intraoperative boost irradiation with kV-photons, a prospective report of acute toxicity in 26 patients treated with hypofractionated whole-breast radiotherapy and intraoperative boost irradiation was published recently. There were no signs of unexpected toxicity [20].

A reduction of overall treatment time of EBRT to 1 to 1.5 weeks by increasing fraction size to a smaller treatment volume can be achieved by accelerated partial breast irradiation (APBI), where data from several phase III trials are available [21–24]. However, in the RAPID trial, shortening overall treatment time to 5 to 8 days by giving two daily fractions of 3.85 Gy was associated with an increased risk of late toxicity and inferior cosmesis [24, 25].

Recently, results from FAST and FAST-Forward, two large randomized controlled trials testing 5‑fraction regimens for adjuvant whole breast radiotherapy, have been published [26, 27]. Due to the large fraction size of 5.2 to 5.6 Gy, we refer to these regimens as ultra-hypofractionation, in analogy to the nomenclature for prostate cancer. This article summarizes the results and provides an overview of potential implications for adjuvant radiotherapy in early breast cancer.

## Results: The FAST and FAST-Forward trials

FAST and FAST-Forward are two randomized controlled phase III trials conducted in the United Kingdom [26, 27]. These trials were built upon the experience with the previous generation of trials on adjuvant radiotherapy for breast cancer, namely the START A and B trials [6–8].

The FAST and FAST-Forward trials were designed in a similar manner as their predecessors. Just as in the START A and B trials, each of the two trials used a three-arm design and compared two different experimental hypofractionation regimens to the standard of care at the time of trial conception. The FAST trial used conventionally fractionated radiotherapy (50 Gy in 25 fractions in 5 weeks) as standard of care while moderately hypofractionated accelerated radiotherapy (40 Gy in 15 fractions in 3 weeks) served as standard of care in FAST-Forward. In the FAST trial, treatment time was kept constant at 5 weeks (just as in START A), whereas FAST-Forward used a very accelerated course of adjuvant radiotherapy over just one week and compared this to the moderately accelerated 3 week standard regimen. Using two slightly different dosages in the experimental arms accounted for possible uncertainties regarding the impact of difference in treatment time. Both trials collected oncological outcomes and toxicity data as well as photographic documentation of normal tissue effects. In addition, the FAST-Forward trial provided a comprehensive assessment of patient-reported outcome.

In the following, we will give an overview of the two individual trials. Important details regarding trial design are listed in Table 1, a summary of results is shown in Table 2. Table 3 gives an overview of the respective fractionation regimens in comparison to conventional fractionation and moderate hypofractionation.

**Table 1:** Trial design for the FAST and FAST-Forward trials

	FAST [27]	FAST-Forward [26]
Timeframe	2004–2007	2011–2014
Sample size	915	4096
Dose/Fractionation	50 Gy/2 Gy/5 weeks 30 Gy/6 Gy/5 weeks 28.5 Gy/5.7 Gy/5 weeks	40 Gy/2.67 Gy/3 weeks 27 Gy/5.4 Gy/1 week 26 Gy/5.2 Gy/1 week
Median follow-up	119.8 months	71.5 months
Primary endpoint	Change in photographic breast appearance	Ipsilateral breast tumor recurrence (non-inferiority margin 1.6%)
Eligibility criteria	pT1–2 (<3 cm) pN0 age ≥ 50 years BCS No chemotherapy	pT1–3 pN0–1 age ≥ 18 years BCS or mastectomy approx. 25% of patients received chemotherapy
Boost irradiation	None	Sequential, 5–8 × 2 Gy approx. 25% of patients

*BCS* breast-conserving surgery.

**Table 2:** Overview of selected outcome data from the FAST and FAST-Forward trials

	FAST (10-year Kaplan–Meier estimates) [27]			FAST-Forward (5-year Kaplan–Meier estimates) [26]		
Ipsilateral breast tumor recurrence	50 Gy	0.7%	–	40 Gy	2.1%	–
	30 Gy	1.4%	HR 1.36 (0.3–6.06)	27 Gy	1.7%	HR 0.86 (0.51–1.44)
	28.5 Gy	1.7%	HR 1.35 (0.3–6.05)	26 Gy	1.4%	HR 0.67 (0.38–1.16)
Any adverse event in the breast/chest wall	50 Gy	33.6%	–	40 Gy	26.8%	–
	30 Gy	50.4%	HR 1.79 (1.37–2.34)	27 Gy	35.1%	HR 1.41 (1.23–1.61)
	28.5 Gy	47.6%	HR 1.45 (1.10–1.91)	26 Gy	28.5%	HR 1.09 (0.95–1.27)
Breast shrinkage	50 Gy	28.5%	–	40 Gy	14.9%	–
	30 Gy	40.5%	HR 1.71 (1.26–2.32)	27 Gy	19.1%	HR 1.34 (1.11–1.62)
	28.5 Gy	33.4%	HR 1.22 (0.88–1.68)	26 Gy	14.6%	HR 0.99 (0.81–1.21)
Breast induration^a^	50 Gy	7.4%	–	40 Gy	2.9%	–
	30 Gy	15.2%	HR 2.22 (1.29–3.84)	27 Gy	6.7%	HR 2.40 (1.63–3.54)
	28.5 Gy	18.6%	HR 2.14 (1.23–3.71)	26 Gy	4.3%	HR 1.42 (0.93–2.17)
Telangiectasia	50 Gy	3.8%	–	40 Gy	3.0%	–
	30 Gy	5.8%	HR 1.55 (0.70–3.45)	27 Gy	4.8%	HR 1.61 (1.06–2.44)
	28.5 Gy	5.5%	HR 1.35 (0.59–3.09)	26 Gy	3.5%	HR 1.41 (0.92–2.16)
Breast/chest wall edema	50 Gy	4.8%	–	40 Gy	5.5%	–
	30 Gy	13.7%	HR 2.98 (1.62–5.48)	27 Gy	10.5%	HR 1.95 (1.47–2.59)
	28.5 Gy	8.6%	HR 1.78 (0.92–3.43)	26 Gy	7.5%	HR 1.36 (1.01–1.85)

Numbers in brackets are 95% confidence intervals.

*HR* hazard ratio.

^a^For FAST-Forward, breast induration at and outside the tumor bed were reported separately. Breast induration outside the tumor bed is shown here.

**Table 3:** Overview of different fractionation regimens used in clinical trials

Regimen	Treatment schedule over the course of 5 weeks	EQD_2_ _Gy_ (α/β = 3.5)
Conventional 25 × 2 Gy	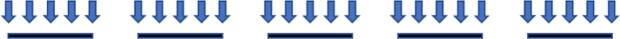	50 Gy
START A 13 × 3.0/3.2 Gy [6]	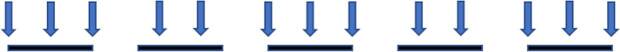	46.1 Gy/50.4 Gy
START B 15 × 2.67 Gy [7]	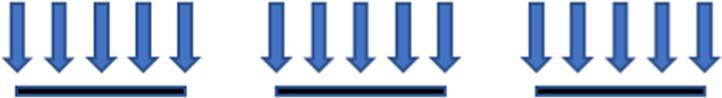	44.9 Gy
FAST 5 × 5.7/6.0 Gy [27]	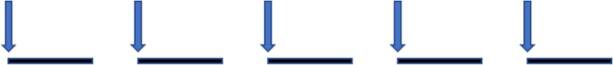	47.7 Gy/51.8 Gy
FAST-Forward 5 × 5.2/5.4 Gy [26]	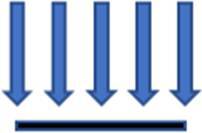	41.1 Gy/43.7 Gy

*EQD*_*2*_ _*Gy*_ Dose equivalent delivered in 2 Gy-fractions without time loss-factor.

The primary endpoint of the FAST trial was change in breast appearance at 2 and 5 years. First results were published in 2011 with a median follow-up of 37.3 months [28]. Moderate or marked changes in photographic breast appearance occurred significantly more often with 30 Gy in 5 fractions as compared to 50 Gy in 25 fractions (risk ratio [RR] 1.7, 95% confidence interval [CI] 1.26–2.29). This was also true when comparing 30 to 28.5 Gy in 5 fractions; however there was no significant difference between 28.5 and 50 Gy (RR 1.15, 95% CI 0.82–1.6). The same pattern—showing inferiority of 30 Gy and comparable results for 28.5 and 50 Gy—was found for late effects such as breast shrinkage, induration, edema and telangiectasia, as well as for a composite endpoint of any moderate or marked adverse effects. Acute adverse events were only assessed in a subgroup of patients. Acute skin reactions grade 3 occurred infrequently but were less common in both experimental arms as compared to the standard arm (50 Gy: 10.9%, 30 Gy: 2.7%, 28.5 Gy: 1.9%).

Long-term results from the FAST trial after a median follow-up of 9.9 years were published recently [27]. To ensure comparability of assessment, 2‑year photographs were re-evaluated along with the 5‑year photographs, yielding a lower number of patients with moderate or marked changes in breast appearance at 2 years (19.1% in the updated analysis vs. 40.5% in the initial analysis). Essentially, results remained unchanged regarding the comparison between treatment arms. Mild and marked changes in photographic breast appearance after 2 and 5 years were significantly more common in patients treated with 30 Gy as compared to 50 Gy (odds ratio [OR] 1.64, 95% CI 1.08–2.49), with a similar trend for 30 Gy compared to 28.5 Gy (no OR provided, *p* = 0.052). There was no significant difference between 28.5 and 50 Gy (OR 1.1, 95% CI 0.7–1.71). Regarding physician-assessed late effects, cross-sectional analysis, longitudinal analysis and time to event analysis (using the Kaplan–Meier method) were presented. While the cross-sectional and the longitudinal analysis (results summarized in Table 2) generally showed inferior results with 30 Gy as compared to 50 Gy and no significant differences between 28.5 and 50 Gy, the Kaplan–Meier analysis revealed a significant absolute increase of 14% for 28.5 Gy compared to 50 Gy for any moderate or marked normal tissue events which was mainly driven by a 6% increase in breast induration (results shown in Table 2). Referral for symptomatic lung fibrosis and ischemic heart disease occurred for only 0.9 and 1.9% of all patients, respectively.

The trial was not powered for statistical comparison of recurrence rates. Estimated cumulative incidence rates for ipsilateral breast events were 0.7% at 5 years and 1.3% at 10 years, corresponding to a total number of 11 local recurrences. There was no statistical difference between the trial arms, albeit with very large confidence intervals due to the low number of events.

First results from the FAST-Forward trial regarding acute toxicity were published in 2016 [29]. This analysis comprised two sub-studies with a total of 350 patients. The percentage of patients with grade 3+ acute skin toxicity according to RTOG criteria was 14% for 40 Gy in 15 fractions, 10% for 27 Gy in 5 fractions and 6% for 26 Gy in 5 fractions for sub-study 1. For sub-study 2, acute toxicity grade 3+ according to CTCAE was 0%, 2.4% and 0%, respectively. Grade 2 toxicity was also more common in the standard arm as compared to the two experimental arms. Of note, the authors argue that RTOG-rated toxicity was considerably higher due to inclusion of pitting edema as grade 3 event. Nevertheless, the acute toxicity grade 3+ rate of 14% in the standard arm is surprisingly high.

Long-term results of the FAST-Forward trial including the primary endpoint of ipsilateral breast tumor relapse were published recently [26]. Median follow-up was 71.5 months. Estimated cumulative incidence rate of ipsilateral breast tumor relapse at 5 years was 2.1% for the standard arm, 1.7% for 27 Gy (hazard ratio [HR] compared to 40 Gy 0.86, 95% CI 0.51–1.44) and 1.4% for 26 Gy (HR 0.67 compared to 40 Gy, 95% CI 0.38–1.16). Non-inferiority was demonstrated for both experimental arms (*p* = 0.0022 for 27 Gy and *p* = 0.00019 for 26 Gy). The absolute numbers of recurrences were low, precluding multivariate analysis. The authors provided the patterns of recurrence and associated clinical parameters in the appendix. There were no significant differences in the risk of any recurrence, disease-free survival and overall survival.

Regarding late normal tissue effects, the patterns observed were similar to the FAST trial. Compared to the standard arm, longitudinal analyses showed that the higher dose experimental arm (27 Gy) displayed a significantly higher risk of almost every reported late normal tissue effect. In the lower dose experimental arm (26 Gy), most marked or moderate normal tissue effects were not significantly different to the standard arm. However, hazard ratios/odds ratios indicated a trend in favor of the standard arm for most of the analyzed items. Regarding breast induration outside the tumor bed and breast/chest wall edema, a significantly higher risk of toxicity was found for the 26 Gy arm compared to the standard arm. An analysis of 3024 patients at 5 years of follow-up reported in the appendix confirmed this and showed a significantly higher risk of breast induration outside the tumor bed with 27 Gy (RR 19.2, 95% CI 2.58–142.9) and 26 Gy (RR 19.1, 95% CI 2.57–141.9) as compared to 40 Gy. Of note, longitudinal analysis in the whole population with assessment of normal tissue effects (3975 patients) which also takes into account earlier evaluations, the odds ratio for moderate or marked breast induration for induration outside the tumor bed was only 2.79 (95% CI 1.74–4.50) for 27 Gy and 1.90 (95% CI 1.15–3.14) for 26 Gy. The absolute risk difference for moderate or marked breast induration outside the tumor bed at 5 years was 2% (1 out of 990 patients for 40 Gy, 20 out of 1008 patients for 27 Gy, 20 out of 1026 patients for 26 Gy), resulting in a number needed to harm of 50 patients. For most late normal tissue effects, the 26 Gy arm was superior to the 27 Gy arm.

Patient-reported outcome data support the above-mentioned findings. Overall, more patients treated with 27 Gy reported moderate or marked events during follow-up. The 26 Gy arm was inferior to 40 Gy only for the item “breast harder or firmer” (OR 1.22, 95% CI 1.00–1.48) with a trend towards superiority with 26 Gy for the item “breast smaller” (OR 0.81, 95% CI 0.65–1.00). In the cross-sectional analysis of patient-reported outcomes, there was an inferiority of 26 Gy compared to 40 Gy only for the item “breast swollen” (RR 2.75, 95% CI 1.17–6.46).

Incidence of symptomatic lung fibrosis and ischemic heart disease was low, without obvious differences between the treatment arms.

While the authors are to be congratulated for these two well-designed and thoroughly conducted trials, there are several limitations that are worth mentioning.

The FAST trial was initially designed to demonstrate a 10% difference of photographic breast appearance at 2 years. The trial was neither powered for a comparison between the three trial arms nor to demonstrate non-inferiority [30]. As previously discussed, reviewing the patient photographs for the 5‑year assessment revealed a significantly lower prevalence of moderate or marked changes in breast appearance than in the previous 2‑year analysis, resulting in a re-evaluation of the 2‑year photographs. Although interpreted by the authors as “likely that perceptions of radiotherapy-related changes changed over the long time period” [27], this may also be seen as a sign of poor reliability and validity of subjective rating of cosmetic outcome based on photographs [30]. The 10-year data for adverse events rely on less than 50% of randomized patients, leading to large confidence intervals for event rates and statistical comparisons. Furthermore, there was a significant increase in the incidence of most normal tissue events with follow-up. The low event rate also leads to large confidence intervals for local recurrence that range from a 70% risk reduction to a more than 6‑fold increase with the 5‑fraction regimens. The FAST trial did only accrue patients with low-risk features without indication for adjuvant chemotherapy and boost irradiation, thus limiting the generalizability of the findings.

The FAST-Forward trial did allow for boost irradiation, which was applied as a sequential boost with 5–8 fraction of 2 Gy. A tumor bed boost was given to all patients under 40 years and to patients aged 40–59 years with adverse risk factors, such as grade 3 and/or lymphovascular invasion. Generally, no boost was given to patients aged ≥ 60 years. The authors reason that it was prudent not to change both fractionation of whole-breast and boost irradiation at the same time and that this was handled in a similar manner in the START trials. Nonetheless, it seems odd to double the overall treatment time to deliver a tumor bed boost in 2 Gy fractions to a much smaller volume. Although patients who had a mastectomy were eligible for the trial, less than 300 patients in each arm were enrolled. Thus, no relevant conclusions can be drawn for this subgroup.

Both trials were not powered for subgroup analysis regarding local recurrence due to the low number of events. Thus, it remains unclear whether the results can be safely applied to all biological and clinical subgroups. Regional nodal irradiation was not permitted in the initial trial design. However, results from a subsequent sub-study of FAST-Forward comparing ultra-hypofractionation to 40 Gy in 15 fractions for patients with an indication for regional nodal irradiation are pending.

## Re-analysis of the FAST-Forward data regarding late toxicity

To analyze the potential impact of follow-up on the outcomes in the FAST-Forward trial, percentages of marked and moderate normal tissue effects (NTE) for the physician-reported endpoints breast distortion, breast shrinkage, induration, telangiectasia, edema, and discomfort were extracted from Fig. A3a, c, e, g, i, k of the supplementary appendix in the FAST-Forward trial publication [26]. The number of patients with moderate or marked NTE (labeled as “quite in bit” and “very much”) were calculated from the extracted percentages and the number of patients at risk at 1, 2, 3, 4, and 5 years follow-up.

**Fig. 1:**
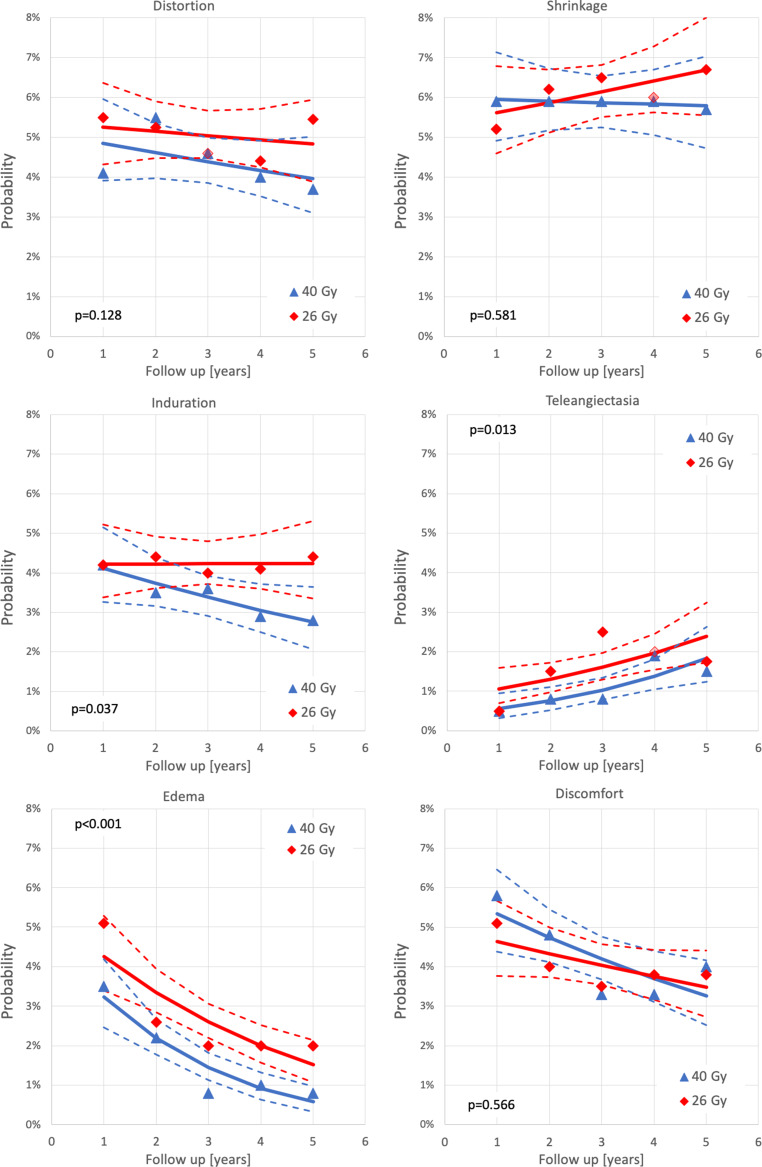
Temporal trends of individual normal tissue effects (*NTE*) in the FAST-Forward trial [26] using binary logistic regression. *Red diamonds* and *blue triangles* represent the extracted data points of the respective end points for 26 Gy in 5 fractions and 40 Gy in 15 fractions, respectively. *Broken lines* indicate the 95% confidence limits of the regression lines and *p*-values the results of the Wald test

**Fig. 2:**
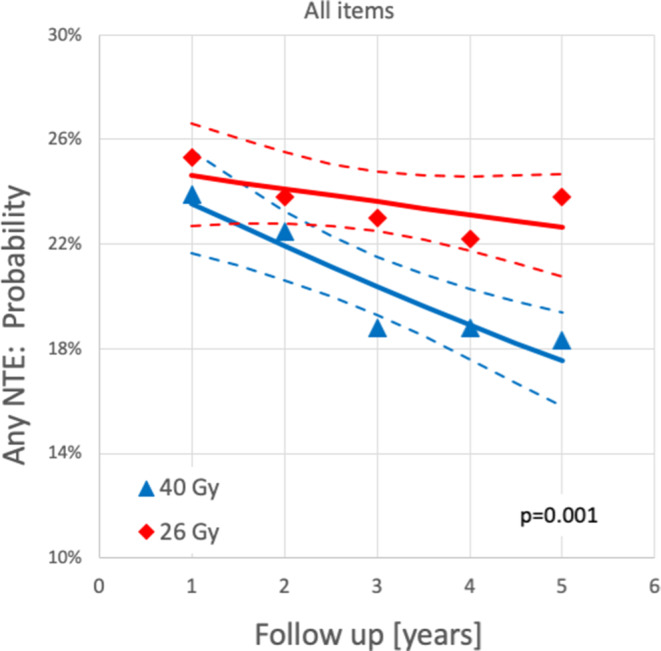
Temporal trends of the sum of all normal tissue effects (*NTE*) in the FAST-Forward-trial [26] using binary logistic regression. *Red diamonds* and *blue triangles* represent the extracted data points of the sum of all NTE for 26 Gy in 5 fractions and 40 Gy in 15 fractions, respectively. *Broken lines* indicate the 95% confidence limits of the regression lines and *p*-values the results of the Wald test

Binary logistic regressions for 40 or 26 Gy were performed separately for all NTE (Fig. 1) and for the sum of all NTE (Fig. 2). Confidence limits of regression lines were calculated based on the covariance matrix. To test for statistical significance between 40 and 26 Gy, a binary logistic regression model including the independent variables time (of follow-up) and group (40 and 26 Gy) was used. All *p*-values were derived from the Wald test. Calculations were carried out with a commercially available software package (SPSS Version 25).

**Fig. 3:**
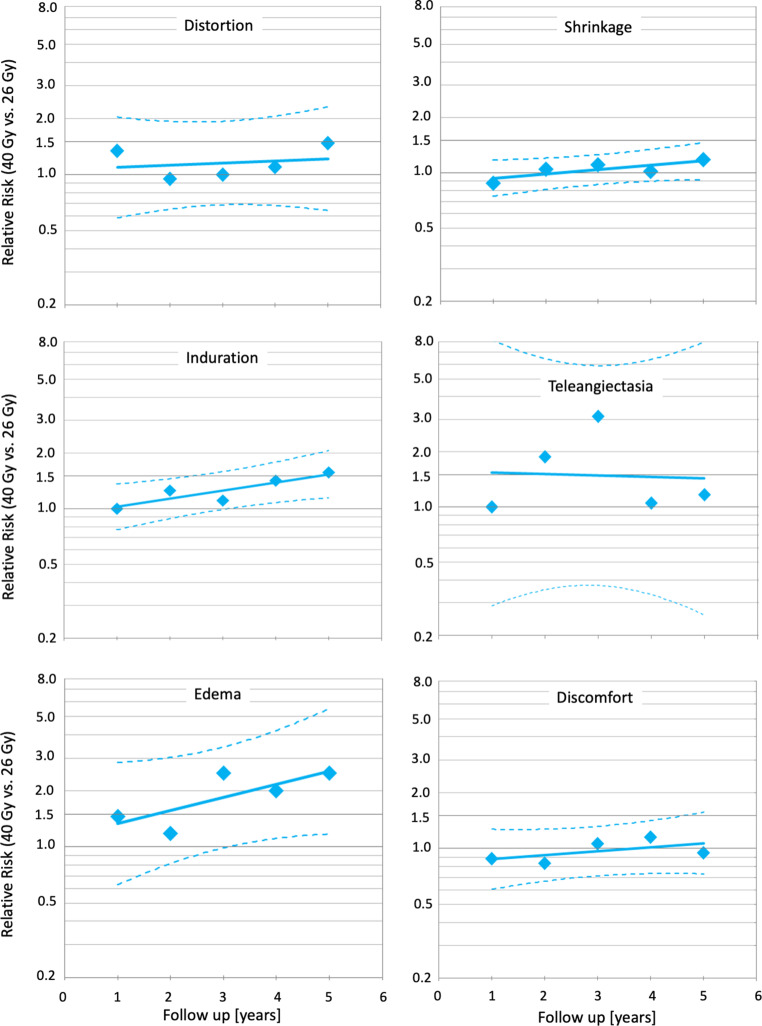
Temporal trends of individual normal tissue effects in the FAST-Forward trial [26] using relative risks. *Diamonds* represent the relative risks (*RR*) for marked and moderate NTE of 26 Gy in 5 fractions compared to 40 Gy in 15 fractions. *Lines* indicates the linear regression and *broken lines* the 95% confidence limits. All *p*-values were derived from the Wald test

**Fig. 4:**
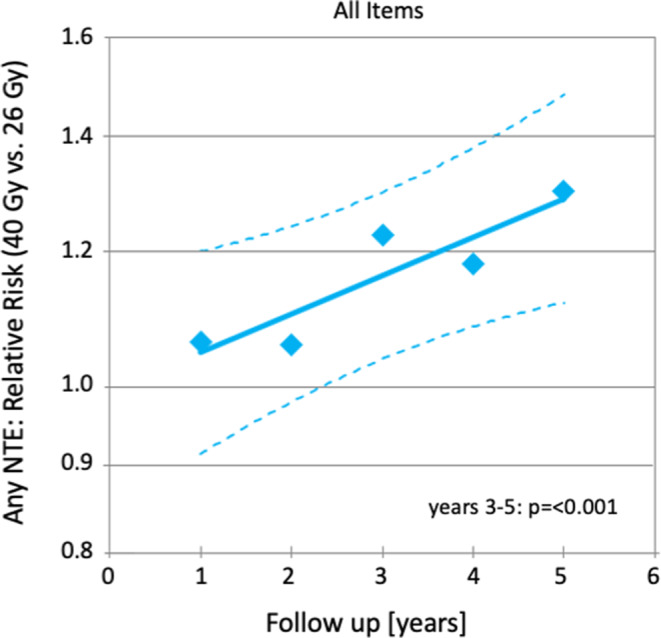
Temporal trends of the sum of all normal tissue effects (*NTE*) in the FAST-Forward trial [26] for relative risks. *Diamonds* represent the relative risks (*RR*) for marked and moderate NTE of 26 Gy in 5 fractions compared to 40 Gy in 15 fractions. *Lines* indicates the linear regression and *broken lines* the 95% confidence limits. All *p*-values were derived from the Wald test

In addition, relative risks for NTE in the 26 Gy arm of the trial compared to the 40 Gy arm were calculated based on the numbers of marked and moderate NTE and the patients at risk at all time points and end points (Fig. 3) including the sum of all NTE (Fig. 4). After logarithmic transformation, linear regressions were carried out for each individual NTE and the sum of all NTE. Confidence limits were calculated by using a standard software package (SPSS Version 25).

Binary logistic regressions showed significant differences in favor of the standard arm for induration, telangiectasia and edema as well as for the sum of all NTE (Figs. 1 and 2). Relative risks of moderate or marked NTE for patients treated with 26 Gy compared to patients treated with 40 Gy increased significantly (*p* < 0.001) over time for all NTE except for telangiectasia (Figs. 3 and 4). The RR of the individual observations only reached significance (*p* < 0.05) after 3 and more years of follow-up for breast induration and edema as well as the sum of all NTE, suggesting that clinically relevant disadvantages may become apparent with longer follow-up.

## Discussion

Adjuvant radiotherapy for breast cancer has undergone a significant evolution during the past decades [31]. Several trials have tried to define a low-risk subgroup of patients who do not benefit from adjuvant whole-breast radiotherapy. However, a significant, although in some trials small, benefit in terms of local recurrence was demonstrated in numerous individual trials and in a meta-analysis [32]. The role of adjuvant whole-breast radiotherapy was further challenged by trials addressing a reduction of the target volume to just the lumpectomy cavity with a clinical safety margin (for review see [33]). Regarding EB-APBI, non-inferiority has been demonstrated in randomized controlled trials for EBRT over 3 weeks [34] and over 1 week [24], while the latter trial showed increased late adverse events and inferior cosmesis [24]. While this was not the case in the NSABP B‑39 trial that included mostly patients treated with the same 1‑week regimen of EB-APBI, equivalence to whole-breast radiotherapy could not be demonstrated for local recurrence and recurrence-free interval was inferior with APBI [23]. The Florence IMRT trial which delivered EB-APBI with 5 fractions of 6 Gy every other day showed similar results to conventionally fractionated whole-breast radiotherapy; however it was not powered to demonstrate non-inferiority [21, 22]. Despite these variations in schedules and outcome, most guidelines have endorsed APBI as a viable option for selected patients with early stage breast cancer with low-risk features [35, 36].

In the meantime, moderate hypofractionation has been adopted as the standard of care for whole-breast radiotherapy based on level 1 evidence regarding its comparable efficacy and tolerability to conventionally fractionated radiotherapy [37]. This is accompanied by an increase in cost effectiveness [38, 39] and patient convenience [4]. After the START trials [6–8] and the Canadian trial [9, 10] defined the standard of care, FAST and FAST-Forward represent the next generation of hypofractionation trials from the renowned group at the Institute of Cancer Research in the UK. The trials were well-designed and conducted in a similar manner to its predecessors with a systematic approach to total dose, fractionation and overall treatment time. Thus, they provide valuable insight into radiation biology of breast cancer as well as clinical results regarding tumor recurrence and late adverse events. Both trials confirm that the α/β-value for breast cancer is in the range of 3.5–4 Gy which is comparable to the α/β-value for most late normal tissue effects (Table 4).

**Table 4:** Estimates for the α/β-value for local control and late normal tissue effects based on randomized controlled trials

Trial	α/β-estimate for local control	α/β-estimate for late normal tissue effects^a^
START pilot [40, 41]	4.0 Gy (95% CI 1.0–7.8)	2.9 Gy (95% CI 1.0–4.8 Gy) for marked change in breast appearance 4.7 Gy (95% CI 1.0–8.6) for breast shrinkage 3.1 Gy (95% CI 1.8–4.4) for breast induration 5.1 Gy (95% CI 1.0–9.5) for telangiectasia 2.3 Gy (95% CI 1.0–4.5) for breast oedema
START A [8]	4.0 Gy (95% CI 0.0–8.9)	3.5 Gy (95% CI 0.7–6.4) for breast shrinkage 4.0 Gy (95% CI 2.3–5.6) for breast induration 3.8 Gy (95% CI1.8–5.7) for telangiectasia 4.7 Gy (95% CI 2.4–7.0) for breast oedema
START A/START pilot-meta-analysis [8]	3.5 Gy (95% CI 1.2–5.7)	Not stated
FAST [27]	Not stated	2.7 Gy (95% CI 1.5–3.9 Gy) for change in photographic breast appearance 2.7 Gy (95% CI 1.9–3.5) for breast shrinkage 1.6 Gy (95% CI 0.0–4.4) for breast induration 3.1 Gy (95% CI 2.3–3.9) for telangiectasia 1.9 Gy for breast oedema^b^
FAST-Forward [26]	3.7 Gy (95% CI 0.3–7.1)	1.7 Gy (95% CI 1.2–2.3) for any moderate or marked clinician-assessed normal tissue effects in the breast or chest wall 2.3 Gy (95% CI 1.8–2.9) for patient-reported change in breast appearance was

*CI* confidence interval.

^a^If not otherwise stated, α/β-estimates are presented for moderate or marked normal tissue effects.

^b^No 95% CI was provided for this estimate.

The important question is: does this constitute a new standard of care? There are several aspects to consider, most importantly tumor control and toxicity.

Regarding tumor control, FAST-Forward demonstrated non-inferiority of 26 and 27 Gy compared to 40 Gy with an appropriate predefined margin of 1.6% [26]. The absolute difference for both arms compared to the standard arm was < 1% and hazard ratios were in favor of the experimental arms. Ten-year local recurrence rates from the preceding FAST trial are based on a total of 11 events [27]. As mentioned above, the FAST trial did not aim at this end point and thus was underpowered for local control comparisons. Hazard ratios in the FAST trial were in favor of the standard arm; however confidence intervals were wide and the absolute excess risk for local recurrence was 0.7 and 1% with 30 and 28 Gy, respectively. In the FAST Forward trial however, the 5‑fraction regimens seem to be comparable to moderate hypofractionation in terms of oncologic outcome, although some uncertainty remains for longer-term results.

Acute toxicity was reduced in both trials with ultra-hypofractionation. This was expected since acute toxicity depends mainly on total dose and less on fraction size. Of note, the acute radiation dermatitis rate in the standard arm of the FAST trial was surprisingly high [28]. In terms of late toxicity, both trials showed an increased risk of late toxicity and inferior cosmesis with the higher dose-regimens of 30 Gy over 5 weeks and 27 Gy over 1 week [26, 27]. This suggests that the dose–response curve for late toxicity is much steeper than for local control. The lower dose-arms (28.5 Gy in 5 weeks and 26 Gy in 1 week) yielded no statistically significant difference for most toxicity items compared to the standard arms, albeit in several items a trend towards inferiority was observed and reached significance for any moderate and marked late effects in the FAST trial and for moderate and marked induration in the FAST-Forward trial. Taking into account all follow-up data concurrently, as done in the re-analysis of FAST-Forward trial described above, the risk of induration, teleangietasia, edema, and the sum of all late NTE was significantly higher in the 26 Gy as compared to the 40 Gy arm (Figs. 1 and 2). Furthermore, in the FAST-Forward trial, the relative risk for any moderate and marked late effects increased over time (Figs. 3 and 4), indicating longer follow-up is necessary to evaluate the long-term safety of this regimen. Interestingly, this trend was not observed in the FAST trial. Thus, the question arises, whether this effect could be a consequence of the drastically shorter overall treatment time in the FAST-Forward trial. Although ultra-hypofractionated radiotherapy in just 5 fractions seem to be safe regarding oncological endpoints, the absolute increase in any moderated and marked late effects in the FAST trial of 14% at 10 years, and in the FAST-Forward trial of 5% at 5 years (Fig. 2) appears to be a relevant long-term burden for our patients compared to 10 additional fractions of radiotherapy over 2 weeks.

Hence, unlike the START B trial, where moderate hypofractionation was unequivocally superior in several subdomains of late toxicity and yielded significantly better oncological outcomes (overall survival and distant metastases free survival) and consequently was adopted as the new standard of care [7, 8], ultra-hypofractionated radiotherapy in breast cancer at this time represents an additional option, but should not be regarded as new standard of care.

Certainly, a shorter course of radiotherapy increases patient comfort [4] and reduces health care expenditure [38, 39]. The reduction in overall treatment time is of relevance especially for patients who are deemed too frail for a 3-week course of adjuvant radiotherapy or in case of other logistical reasons precluding the use of moderate hypofractionation. Use of ultra-hypofractionation may be preferentially considered for patients not requiring a tumor bed boost. Nonetheless, even with a sequential conventionally fractionated tumor bed boost, overall treatment time is still reduced by two weeks compared to moderate hypofractionation. This advantage would shrink to one week with utilization of moderate hypofractionation with a simultaneous integrated boost or a preceding intraoperative boost. As mentioned before, definitive results from randomized controlled trials evaluating these regimens (RTOG 1005 [NCT01349322], HYPOSIB [NCT02474641] as well as the TARGIT‑B trial [NCT01792726] and the prospective single arm HIOB trial [NCT01343459]) are pending.

Especially in light of the current COVID-19 pandemic however, the reduction in ambulatory visits and thus, a reduced risk of virus transmission together with a lower utilization of health care resources may be critical arguments to endorse ultra-hypofractionation for breast cancer [42, 43].

## Conclusions

Based on the results of FAST and FAST-Forward, adjuvant whole-breast radiotherapy in 5 fractions should be used with caution in patients with a favorable long-term prognosis. However, it may be regarded as an additional option in the radiation oncology armamentarium, especially in elderly frail patients and in settings with limited health care resources. Nevertheless, in light of the excellent results of adjuvant breast cancer treatment nowadays, the bar is set high and a reduction in overall treatment time of two weeks should generally not be the only motivation to adopt a new standard of care. Tumor control and toxicity remain pivotal in the consideration of treatment options. Thus, shared-decision making regarding ultra-hypofractionated whole-breast radiotherapy in 5 fractions should include a discussion of residual uncertainties regarding long-term tumor control and a potential increase in late toxicity. To date, ultra-hypofractionated radiotherapy should not be used in patients who underwent mastectomy or who require regional nodal irradiation. Furthermore, in the absence of further data, caution is advised in young patients and patients with connective tissue diseases.
